# Degraded Time-Frequency Acuity to Time-Reversed Notes

**DOI:** 10.1371/journal.pone.0065386

**Published:** 2013-06-17

**Authors:** Jacob N. Oppenheim, Pavel Isakov, Marcelo O. Magnasco

**Affiliations:** Laboratory of Mathematical Physics, Rockefeller University, New York, New York, United States of America; UNLV, United States of America

## Abstract

Time-reversal symmetry breaking is a key feature of many classes of natural sounds, originating in the physics of sound production. While attention has been paid to the response of the auditory system to “natural stimuli,” very few psychophysical tests have been performed. We conduct psychophysical measurements of time-frequency acuity for stylized representations of “natural”-like notes (sharp attack, long decay) and the time-reversed versions of these notes (long attack, sharp decay). Our results demonstrate significantly greater precision, arising from enhanced temporal acuity, for such sounds over their time-reversed versions, without a corresponding decrease in frequency acuity. These data inveigh against models of auditory processing that include tradeoffs between temporal and frequency acuity, at least in the range of notes tested and suggest the existence of statistical priors for notes with a sharp-attack and a long-decay. We are additionally able to calculate a minimal theoretical bound on the sophistication of the nonlinearities in auditory processing. We find that among the best studied classes of nonlinear time-frequency representations, only matching pursuit, spectral derivatives, and reassigned spectrograms are able to satisfy this criterion.

## Introduction

**Figure 1:**
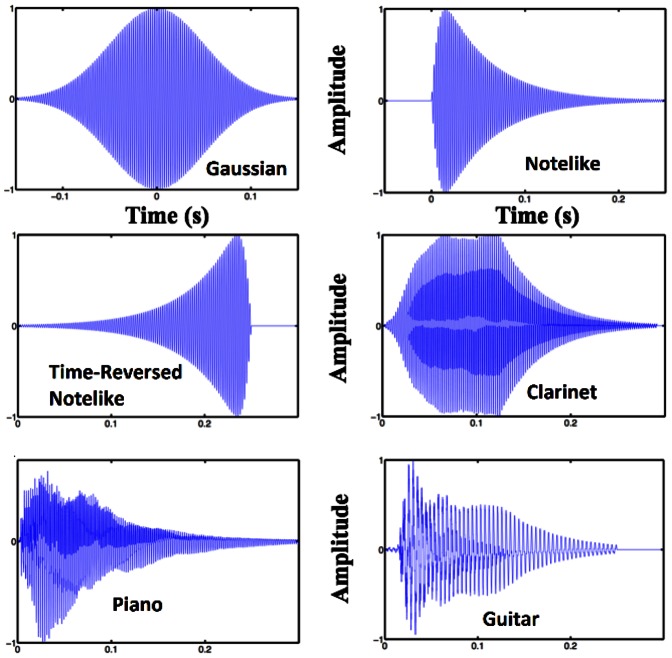
Wavepackets used in experiments and musical notes, at 440 Hz. Musical notes are shown for comparison with our test stimuli as well as to demonstrate the waveforms of certain types of natural sounds. Note that each of the musical notes is time-reversal symmetry broken.

It has long been proposed that the human auditory system is in some way “optimized” for natural sounds. Ecological theories of perception suggest that hearing evolved to detect the sounds necessary for survival and successful reproduction. Such sounds fall into three groups: those of conspecifics, heterospecifics (predators and prey of humans), and the elements. This principle is seemingly obeyed in the case of the range of frequencies and volumes that humans can hear. It has been suggested that the auditory system had evolved to optimally encode natural sounds [1]. Much recent work in this direction has involved examining the output of neurons in an animal subjected to auditory signals both with natural and unnatural statistics, in amplitude [2], [3], spectrum [4]–[6], and scale-invariance [7]. While these studies focus on the higher order properties of natural sounds, such as the energy in various spectral bands, comparatively little attention is paid to the lower order properties of such sounds. Attias and Schreiner noted that the frequency and amplitude spectra of a broad class of sounds, ranging from wolf vocalizations to symphonic music, occupy a limited region of parameter space [8]. Both con- and hetero-specific sounds are nearly always time-reversal symmetry broken, composed of elements with sharp attacks and long decays [7]. Vocal production by animals [9] as well as the noises caused by their movements, such as a tiger stepping on a twig, or a deer rustling the branch of a tree, are time-reversal symmetry broken, for reasons grounded in the physics of energy dissipation. This effect may be not be obvious on spectrograms, as the onset of many notes is so rapid, and the sustain and decay so elongated, that the waveform appears to be a continuous block of sound. However, from the perspective of quantifying the possible envelopes of notes, such sounds correspond to a rather small region of parameter space. One need look no further than the utility and ubiquity of gammatones in scientific research and the ADSR (attack-decay-sustain-release) description of notes in synthesizers. The second row of Figure 1 demonstrates this property for three different methods of sound production, a clarinet, a piano, and a guitar.

The shape of a sharp attack followed by a long decay (or sustain-release) reflects the physics of sound production for a broad class of natural sounds - a class that includes many sounds likely to be important for human survival in the wild. A burst of energy is produced and decays due to viscous damping: a mallet hits a drumhead (or an animal steps on a branch), a burst of air is forced through a trumpet (or the syrinx of a bird), a string (or a branch) is plucked and released [7]. Attempts to reconstruct the receptive fields of auditory neurons using the reverse-correlation method have found filters shaped like stylized versions of the above examples [10]. This is the result we would expect on the grounds of optimal-information transfer: statistical priors of a rapid-attack, slow-decay form. The reverse-correlation method, however, has only a limited ability to reconstruct auditory filters, even in the case of simulated data [11]. Additionally, such spectral methods are thrown into doubt by the existence of essential nonlinearities in the cochlea [12], [13] and the recent results that human auditory perception is more precise than any linear (spectral) method can account for [14].

Unlike other “higher-order” properties of natural sounds, such as amplitude fluctuations and correlations within the spectrogram, the ability of the auditory system to respond to stylized time-reversal symmetry broken notes versus unnatural ones can be easily tested using standard psychophysical methods. Our previous work had involved the creation of a protocol for measuring simultaneous human time-frequency acuity. This methodology can be adapted to test how both time and frequency acuity change with variations in the envelope of the notes presented. Such a test would be able to lend credence to the existence of time-reversal symmetry broken statistical priors in the auditory system.

Time-reversal symmetry breaking has been found in the response of cortical neurons to an animal's own song, but not a time-reversed version of it in species such as Marmosets [15] and White-Crowned Sparrows [16]. Similar assays are frequently used to determine selectivity for an animal's own vocalizations versus those of conspecifics [17]. These studies additionally shed light on issues of temporal versus rate coding; in both cats and guinea pigs, the information content of spike patterns in neurons has been shown not to differ much between the “time-forwards” and “time-reversed” versions of conspecific vocalizations, however, the temporal spiking pattern differs strongly [18]. In one experiment, ferrets were trained to distinguish between forward and reversed versions of marmoset calls: spike data indicated a much greater degree of synchrony in spike patterns for the “time-forward” version [19], suggesting perhaps enhanced temporal acuity to “time-forward” vocalizations.

Time-reversal symmetry broken statistical priors would help to explain the utility of the numerous nonlinearities found in the auditory system, which were previously shown to be essential to the acuity of human hearing [14]. A difference in acuity between a note and its time-reversed form would constrain mathematical descriptions of auditory processing. Our goal was thus two-fold, to see if the auditory system is primed to better process naturalistic, time-reversal symmetry broken notes, and to use such psychophysical data to better understand the nonlinearities present in the auditory system.

## Methods

We used the same testing procedure as in [14] to test for simultaneous time-frequency acuity. The relevant specifications of equipment, training tasks, experimental parameters, and preliminary data fitting for the extraction of physiological parameters may be found therein. What follows is a brief overview of the experimental procedure with emphasis on the adaptations of our original protocol to investigate the effects of time reversal.

### Human subjects

Our work was a continuation of a prior study [14], approved by the IRB under Rockefeller University protocol MAG-0694, approved initially for the period 2/1/2010–1/13/2011 and renewed annually since. As in the previous work, we enrolled highly-musically-trained subjects, due to their superior and stable performance on simultaneous time-frequency tasks. Composers and conductors especially were distinguished their better ability to parse out distractions and maintain focus. Subjects were recruited by means of IRB-approved fliers and word of mouth among New York City conservatories. Written informed consent was taken from all subjects. This study consisted of 12 subjects, many of whom were students of composition or conducting [14]. Our sample is clearly not representative of the population at large. Preliminary testing had revealed that musicians, especially composers and conductors, had consistent performance on our psychophysical tasks. In order to fit a psychometric curve [see below] and extract properly defined acuities with the minimum of error, we desired subjects whose performance would not waver over a full set of tests (approximately 90 minutes). While naive subjects perform nearly as well in frequency or timing as musically trained ones, their performance will start to falter after 5–10 minutes of testing. Once they are asked to either ignore a distractor note or measure time and frequency simultaneously [see below], their performance became substantially worse. These observations imply that musical training does not enhance acuity, but rather aids subjects in performing consistently well in the unnatural setting of psychophysical tasks. We additionally desired results directly comparable with previous work involving the same basic auditory task [see below].

### Stimuli and Tasks

Three types of wavepackets were used: a Gaussian with a width of 0.05 seconds, a “notelike” envelope that approximated a musical note with a rapid increase proportional to 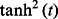 followed by a slower exponential decay, and a time-reversed version of the second pulse, that is, a gradual attack followed by a sharp decay, see Figure 1. The width of the Gaussian was used as the time constant of the exponential in the “notelike” and “time-reversed” pulses. We extracted the theoretical uncertainties in time and frequency of each pulse by integration in MATLAB (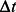 and 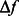); these are the analogous quantities to those extracted from the fitted psychometric curve (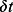 and 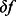), see below. Testing was performed at 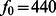 Hz, as this was shown in earlier work [20] to yield optimal performance on the uncertainty task. The flanking note was separated from the center by a factor 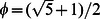, the least harmonic interval, minimizing interference effects.

### Tasks and testing sequence

We performed the auditory equivalent of a two-dimensional Vernier task, in which a frame is given specifying a horizontal (time) and a vertical (frequency) direction, and the test note is misaligned from this frame. Four training tasks were given, including basic frequency and time discrimination tasks with and without a distractor note. Subjects performed 5 sets of 20 questions each per task, with additional sets of 20 if necessary for convergence of data, or to eliminate poor initial performance on a task. All tasks adapted dynamically to the subject's performance according to the two down, one up paradigm. The difficulty of each task (the amount the test note was misaligned from the flanking notes), 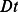 and 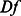, was chosen from a Gaussian distribution. As in our prior work, we adjusted the variance of this distribution, increasing it by 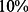 after two correct responses and decreasing it by 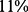 after an incorrect response. Simulations showed these update rules to give the most even sampling of the “steep” region of the psychometric curve and yield rapid convergence of parameters. We set up both subtasks as a 2AFC (two alternative forced choice) asking whether the test note comes before or after the high note, and is above or below the first note in pitch, testing for time and frequency acuity simulteously. We fit a psychometric function of form erfc((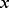-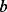)/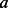), implicitly assuming that the probability of not noticing a difference was normal in the difference. The parameter of this error function, 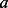 is directly interpretable as a statistical “uncertainty” or standard deviation, giving a well-defined measure of the limens of discrimination for frequency and timing. These value are termed 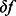 and 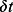, respectively, below.

### Data Analysis

In theory, we should be able to use an F-test as the acuity values we use are the second moments of the Gaussian PDFs for the probability of a certain subject making an error on a certain task (time or frequency). When we fit a psychometric curve, we fit the CDF of this distribution. Our assumption, one that is standard in psychophysics, is that this CDF can be modeled as an error function, which is justified in depth in the supplement of [14]. As the CDF is an error function, the PDF is Gaussian. The second moment of a sample of random variables drawn from a Gaussian distribution is easily shown to be 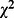. We could then compare the ratio of acuities from the same subject on different tasks, which, if the tasks were the same would yield an 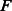-distribution. However, our data consists of binary values, measured at time and frequency points chosen by us, rather than time and frequency points chosen from the PDF. Hence, the effective number of data points, which determines the number of degrees of freedom of the relevant 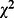 and F distributions are not well defined. We thus chose to use a nonparametric, bootstrap based test [see below].

## Results

### Psychophysical Measurements

**Figure 2:**
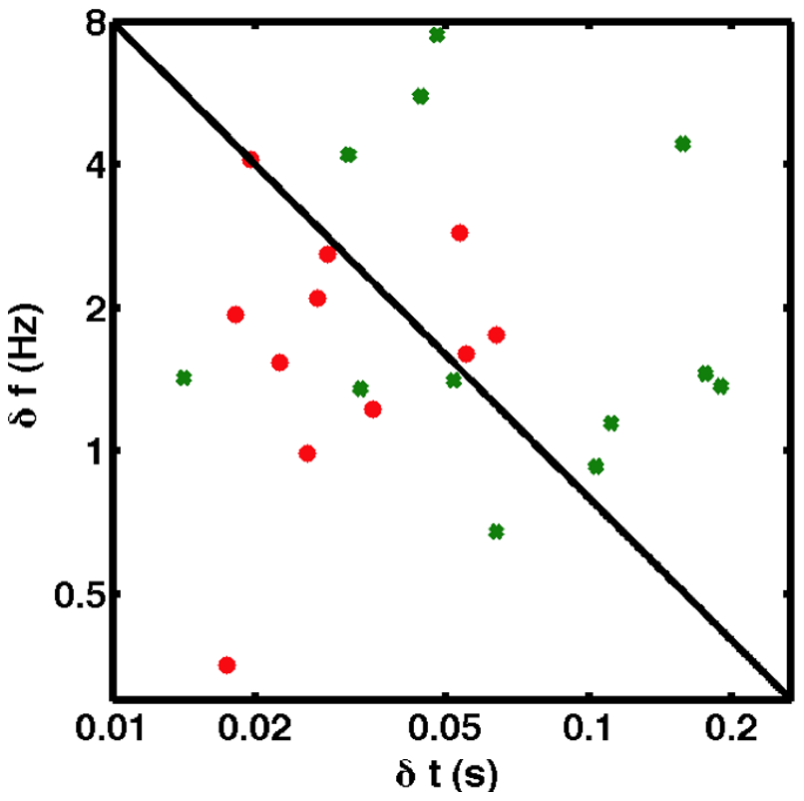
Results of Psychophysical Testing. We plot our data on a standard time-frequency plane with 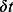 on the x axis and 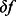 on the y axis. The red circles are the results from the “time-forward” notelike pulse, the green crosses are from the “time-reversed” version. The uncertainty bound is the black diagonal; the axes are logarithmic. The data from the “time-reversed” pulse are shifted notably to the right (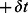) from the original “time-forward” notelike one.

**Figure 3:**
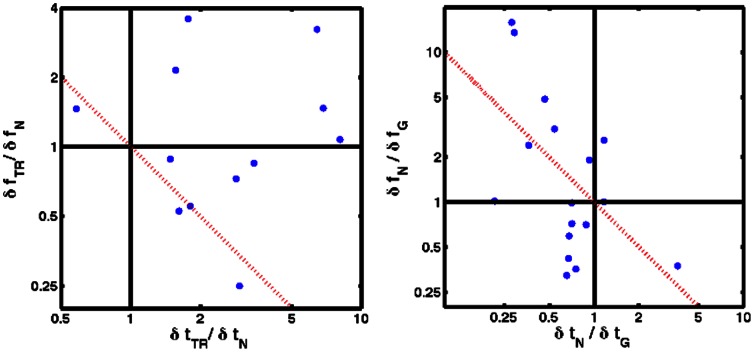
Difference in performance on different shaped notes. On the left is performance on the “time-reversed” pulse divided by performance on the “time-forward” notelike pulse. On the right is performance on the “notelike” pulse divided by performance on the gaussian pulse. The dotted red line indicates a perfect tradeoff in performance, i.e. 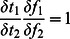. On both figures, axes are logarithmic.

Upon testing subjects with all three pulses, it was immediately apparent that performance on the time-reversed task was notably worse. While several subjects managed relatively similar performance on the preliminary frequency and time discrimination tasks, comparing the notelike (subscript N) and the time-reversed pulses (subscript TR), no one managed better, or even similar performance on the combined time-frequency discrimination task. In Figure 2, we plot our data on a time-frequency plane with logarithmic axes: red dots indicate results from notelike pulses; green x's, time-reversed. Note that the data from the time-reversed pulses seem to cluster on the right-hand side of the plot. We examined the vector of change in performance between the notelike and the time-reversed notelike pulses (Figure 3, left-hand side). Note the dramatically worse performance in timing, frequency, or both. A compensatory improvement (e.g. an improvement in timing acuity balancing a decay in frequency acuity) occurred in timing acuity in only two subjects and of relatively small multiplicative effect, whereas an improvement in frequency discrimination was seen in four subjects.

We tested the significance of the change in frequency and timing acuity, as well as the uncertainty products as a measure of total acuity, using the bootstrap distribution of the acuity values. We generated 1000 bootstraps for each task 5, notelike dataset. We then fit these resampled datasets and extracted the parameter of the fitted error function (the acuity values). Using this distribution, we calculated at what quantile the performance on the time-reversed notelike pulse would fall, allowing us to calculate the probability that the subject's acuity on the “time-reversed” notelike pulse was the same as on the “time-forward” version. Unless otherwise noted, 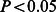 indicates statistical significance below.

For 11 of the 12 subjects we observed a fall in timing acuity (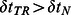); 7 were significant (4 with 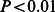), though all 11 were significant with 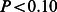. Two subjects had a significant decrease in frequency acuity (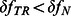), whereas 5 had a significant increase in acuity (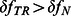). We may thus conclude that the strong change in timing acuity apparent in Figure 3 is real, while the change in frequency acuity seems to be a combination of statistical fluctuations and a decrease for some subjects. Total acuity was significantly worse for 5 subjects and approximately the same for the other 7, albeit in 5 cases there was an insignificant increase in total acuity.

As a methodological control, we combined our data with that of our previous study [14] and examined the difference in simultaneous time-frequency acuity between gaussian and notelike pulses (Figure 3, right-hand side). In our previous work, we had found nearly identical total acuity, as measured by the uncertainty product, for both of these pulses, but with better timing-acuity in the “notelike” ones. Out of 17 pairs of tests, 12 subjects showed improved performance in timing and 8 in frequency. We applied the same bootstrap-based test, comparing the “time-forward” notelike acuities with the bootstrap distribution of the Gaussian data. We found a significant decrease in 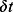 in 6 of 17 cases and a decrease in 14 total of the 17. This improvement in timing acuity was balanced by a decrease in frequency acuity (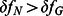) in 7 cases and no change in the 10 others. The total acuity was significantly improved in 2 cases; it remained unchanged in the other 15, again qualitatively consistent with the results displayed in Figure 3.

It is worth noting that these results should not be affected by our use of musically trained subjects. As we remark above (and in the supplement to [14]), musically trained subjects were chosen for the consistency of their performance, not for their possibly enhanced acuities. Consistent values for time and frequency acuities have allowed us to precisely measure the difference in response to time-forward and time-reversed notes, a measurement that would be considerably more difficult in naive subjects.

### Implications for Models of Auditory Processing

Many nonlinear time-frequency distributions may be viewed as modifications of Fourier analysis. They are thus blind to the arrow of time and obey a modified uncertainty relation, changing only the constant on the right hand side of the canonical Fourier Uncertainty Principle: 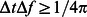, where 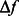 and 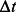 are the standard deviations or “uncertainties” of the signal in frequency and time, respectively [20]. For a further discussion of these quantities and their psychophysical analogues see [14]. If we naively assume that human hearing is limited by such a modified bound, we may estimate where such a bound could lie from the “theoretical uncertainty” (the standard deviations) of the notes presented, here 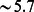 times the optimal value (a Gaussian) and the performance of the best subject 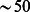 better than the Fourier limit and then extract the maximum such prefactor that could explain our results, and the “order” of the minimum nonlinearity necessary.

We may define a hierarchy of nonlinear time-frequency distributions based on the order of their nonlinearity: the number of the copies of the signal that are convolved in the transformation. As there is no reason to believe a general closed form exists for arbitrary pulses, we focus below on Gaussian wavepackets. For mathematical simplicity, we work with the angular frequency, 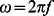. All time-frequency representations may be written in terms of Cohen's class [20]:
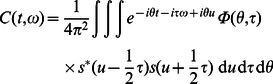
(1)Here, 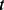 and 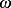 are our time and frequency variables in the final representation, 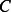; 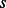 is the original signal, 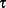 the running window in time, centered at 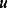, and 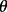 the frequency window. 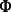 is denoted the kernel of the transformation. For all bilinear time-frequency representations (Cohen's Class), 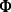 is independent of 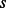. The simplest member of this class is the Wigner Distribution, for which 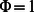. A signal-dependent kernel changes the order of the distribution. One may recover the spectrogram by inserting a window-dependent kernel and writing 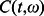 as a square. To obtain properly-construed marginal distributions in time and frequency and thus values for 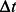 and 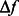,
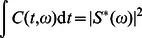
(2)

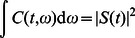
(3)we must have 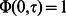 and 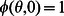. Hence, any time-frequency representation whose kernel obeys the above conditions must obey a modified uncertainty principle of the form 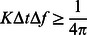, with K some constant, dependent on both the wavepacket shape and the enveloped frequencies.

To measure the increase in precision, we evaluate the Cohen's class integrals for gaussian-enveloped pure tones, 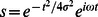, both because of their optimality in the linear case and our data on such packets. For 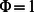 (the Wigner Distribution), we have after taking the Fourier transform of 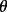 and integrating out 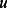,
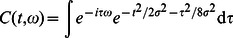
(4)Without evaluating this integral, we may read off 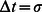 and 
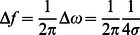
, a factor of two improvement over linearity. Examining the general case more carefully, we see that the origin of improved performance is due to the convolution of the signal with itself. Without any prior information, 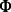 is not a function of 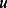, 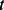, or 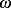. The kernel is only able to affect the uncertainty in the frequency domain by action on 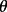. In the case of Gaussian wavepackets, where enhanced precision arises from the largeness of N in the Fourier transform of 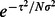, the only kernel that could improve upon the Wigner distribution would be of the form 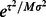 with 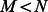. Such a kernel, by privileging large 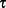
's over small ones would be catastrophic in the case of multiple signal components, amplifying the destructive interference of the Wigner Distribtuion. The kernels used in time-frequency analysis tend to be sharply peaked to decrease this interference. A paradigmatic example is the Choi-Williams Kernel, 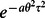 which applies a Gaussian window in both time and frequency to the signal autocorrelation, thus reducing the enhanced precision in the frequency domain from convolving the signal with itself.

We may apply the same logic to kernels that are explicitly dependent on the signal itself. Each additional pair of the signal, 
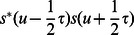
, will build higher order correlations into 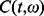, increasing precision by a factor of 2. Any other terms will be used to suppress interference (increasing resolution) and cannot add to precision. Our best subject was able to beat the Uncertainty Principle by a factor of 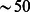, suggesting at a minimum, a 12th order nonlinearity (6 factors of 2, each arising from a signal-conjugate pair) is necessary to explain human auditory perception. Such a bound rules out the traditional Cohen's Class representations and throws into doubt the Hilbert-Huang method and other PCA-based methods, which, involving a matrix-inversion, can be estimated to be of greater than 3rd but less than 12th order [21]. Our results thus suggest that only matching-pursuit, the multi-tapered spectral derivatives [22], [23] and the reassigned spectrograms [24]–[28] can hope to capture the precision of the auditory system.

## Discussion

The significant increase in timing acuity unaccompanied by a drop in the total acuity for a pulse with considerably larger variances in timing and frequency indicates that either the precision of human time-frequency perception operates in a realm distant from the true uncertainty bound, or such a bound does not exist for the auditory system. We may increase both the physical standard deviations (uncertainties), 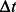 and 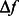 of a note, shaping the envelope to aid in temporal perception, and find improved timing acuity without a decrease in simultaneous time-frequency acuity. Examining Figure 3, in the right panel, we see data both above and below the line 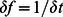, which represents a perfect tradeoff between time and frequency. In the right panel, we see that the best performers fall almost exactly on this line, indicating a perfect tradeoff between time and frequency acuity when going from “notelike” to time-reversed pulses; every other subject is above this line. The results of these subjects, who have statistically significantly worse time-frequency acuity to “time-reversed” notes, indicate degraded acuity to time-reversal symmetry broken notes for one direction of the arrow of time (backwards in this case).

The indifference of the Fourier Transform, a naive, but frequently default view of signals analysis, to the arrow of time is not reproduced in perceptual acuity. A weaker version of time-reversal symmetry might suggest that overall time-frequency acuity for notes is the same in both the time-forward and time-reversed cases, with increased temporal acuity in the “time-forwards” direction trading off with increased frequency acuity in the “time-reversed” direction, corresponding to focussing on the better defined feature of the first part of the note in time-frequency space. While some subjects are able to manage this feat to a certain extent, it is by no means uniform across subjects. Compensatory improvements in frequency perception may not explain all of their performance; in many subjects we have noticed an increase in performance on the simultaneous time-frequency discrimination task with exposure for the first 2–3 trials. It is likely this is the origin of the improved timing perception of the one subject who could distinguish notes in time better in the time-reversed case than the original one. To properly disentangle which effects are due to repeated exposure and which are due to innate statistical priors in the auditory system—those which are built from years of exposure to time-reversal symmetry broken sounds and those which are due to the constraints of auditory physiology—requires further study and careful controls for task learning.

We are agnostic as to when the time (or frequency) of a note is determined by the brain, be it prospectively (when it is first noticed), or retrospectively (after post-processing in the CNS). There is some evidence that the timing of a note is determined after the note has finished, at least for musical notes [29]. Several of our subjects would occasionally take up to 15 seconds to respond after a stimulus on Task 5, suggesting a high degree of post-processing, possibly in the form of “playing” the stimulus back in their head. It is important to note, however, that our mathematical results are unaffected by when time and frequency measurements are made by the auditory system. Mathematically, all that matters, is that the subject hears the stimulus once and is asked to discriminate the notes in time and frequency.

We have demonstrated that human auditory perception is enhanced for notes with a sharp attack followed by a long decay, corresponding to the physics of production of a large and the morphology of a large and ecologically relevant class of natural sounds. We have used simple, direct psychophysical measurements to test for the changes in simultaneous time-frequency acuity after reversing the temporal direction of symmetry-broken pulses, lending credence to the existence statistical priors for sharp attack, long decay sounds. Such results add to the growing body of evidence that human auditory processing is adapted for natural sounds. Not only then is auditory processing inherently nonlinear, these nonlinearities are seemingly used to improve perceptual acuity to sounds that correspond to the physics of natural sound production.

Our experimental results inspired a look at the hierarchy of nonlinear time-frequency distributions and allowed the placement of a lower bound on the degree of such a nonlinearity, ruling out many of the simplest and most frequently used nonlinear time-frequency representations, the bilinear ones of Cohen's Class, as well as suggesting that ones based on PCA, such as Hilbert-Huang are not of high enough order to account for our data. Among those that remain, matching pursuit, spectral derivatives and the reassigned spectrograms, we hope that further psychophysical measurements as well as considerations of the ability to implement such algorithms in neural “hardware” may further narrow the class of plausible methods of auditory processing. Lastly, our observations about time-reversal symmetry breaking and the temporal precision of the auditory system suggest further research into this ecologically-relevant domain. The time at which a note is deemed to occur is poorly understood; a “leading-edge” detector model for temporal acuity could help explain these results.
